# Stereotactic body radiotherapy for lung metastases as oligo-recurrence: a single institutional study

**DOI:** 10.1093/jrr/rrv063

**Published:** 2015-10-22

**Authors:** Masahiko Aoki, Yoshiomi Hatayama, Hideo Kawaguchi, Katsumi Hirose, Mariko Sato, Hiroyoshi Akimoto, Hiroyuki Miura, Shuichi Ono, Yoshihiro Takai

**Affiliations:** Department of Radiology and Radiation Oncology, Hirosaki University Graduate School of Medicine, 5 Zaifu-cho, Hirosaki, Aomori, 036-8562, Japan

**Keywords:** stereotactic body radiotherapy, oligo-recurrence, oligometastasis, lung tumor

## Abstract

The purpose of this study was to investigate clinical outcomes following stereotactic body radiotherapy (SBRT) for lung metastases as oligo-recurrence. From May 2003 to June 2014, records for 66 patients with 76 oligo-recurrences in the lungs treated with SBRT were retrospectively reviewed. Oligo-recurrence primary sites and patient numbers were as follows: lungs, 31; colorectal, 13; head and neck, 10; esophagus, 3; uterus, 3; and others, 6. The median SBRT dose was 50 Gy (range, 45–60 Gy) administered in a median of 5 (range, 5–9) fractions. All patients received SBRT, with no acute toxicity. Surviving patients had a median follow-up time of 36.5 months. The 3-year rates of local control, overall survival and disease-free survival were 90.6%, 76.0% and 53.7%, respectively. Longer disease-free interval from initial treatment to SBRT, and non-colorectal cancer were both associated with favorable outcomes. Disease progression after SBRT occurred in 31 patients, most with distant metastases (*n* = 24) [among whom, 87.5% (*n* = 21) had new lung metastases]. Among these 21 patients, 12 were judged as having a second oligo-recurrence. Additional SBRT was performed for these 12 patients, and all 12 tumors were controlled without disease progression. Three patients (4.5%) developed Grade 2 radiation pneumonitis. No other late adverse events of Grade ≥2 were identified. Thus, SBRT for oligo-recurrence achieved acceptable tumor control, with additional SBRT also effective for selected patients with a second oligo-recurrence after primary SBRT.

## INTRODUCTION

Distant cancer metastases help define advanced stage disease and may form as a result of hematogenous metastases of cancer; it often indicates a poor patient prognosis and a limited mean life expectancy. Although systemic chemotherapy and/or molecular targeted therapy have been administered to patients with distant metastases, most patients treated with such systemic therapies are considered to have an incurable disease.

In contrast, some patients have distant metastases in only a few regions. In 1995, Hellman and Weichselbaum proposed an intermediate state of metastasis termed ‘oligometastasis’, in which the number and sites of metastatic tumors are limited [1]. The lungs are among the common sites of metastasis following radical treatment of a primary cancer. Although surgical removal is considered to be a radical treatment for patients with lung oligometastases, many patients are considered to be medically inoperable. Stereotactic body radiotherapy (SBRT) is considered to be an alternative treatment for lung oligometastases and has been widely used to treat patients with lung oligometastases worldwide [2].

Recently, Niibe *et al.* addressed the states of oligo-recurrence, in which the patient shows one to several distant metastases/recurrences in one to several organs, and disease control at the primary cancer site [3, 4]. Long-term survival can be expected for patients with lung oligo-recurrence. However, the rules for radiotherapy and the prognostic factors for lung oligo-recurrence have not yet been fully elucidated. In this study, we retrospectively reviewed SBRT outcomes for patients with oligo-recurrence in the lungs and evaluated survival, patterns of failure, and treatment-associated toxicities.

## MATERIALS AND METHODS

### Eligibility criteria

The eligibility criteria for this study of SBRT for oligo-recurrence in the lungs were as follows: (i) one to three lung metastases, (ii) locally controlled primary cancer, (iii) tumor diameter ≤5 cm, and (iv) no other distant metastases/recurrences.

### Patient and tumor characteristics

From May 2003 to June 2014, 241 patients, including those with primary lung cancer and lung oligo-recurrence, underwent SBRT at our institution. A total of 66 patients [44 men and 22 women; median age, 71 (range, 27–87) years] with 76 lung oligo-recurrences fulfilling the study eligibility criteria and treated with SBRT were retrospectively reviewed. The primary sites for oligo-recurrence in a number of patients with metastatic lesions at that site were as follows: lungs, 31; colorectal, 13; head and neck, 10; esophagus, 3; uterus, 3; and others, 6. Histological or cytological confirmation of oligo-recurrence in the lungs was required by biopsy or cytological confirmation for the 31 patients with 33 tumors. The results showed 24 adenocarcinomas and 9 squamous cell carcinomas. Although 35 patients with 43 tumors were not confirmed histologically by bronchoscopy and/or CT-guided biopsy, these were diagnosed as oligo-recurrence clinically because of increasing tumor size, increasing tumor markers, and positive accumulation of tracer on ^18^F-fluorodeoxyglucose (^18^F-FDG) positron emission tomography.

All primary cancers were controlled by initial treatment; the initial treatments for the primary cancers comprised surgery for 52 patients and definitive radiotherapy for 14 patients. A median disease-free interval (DFI) from initial treatment to SBRT for oligo-recurrence was 31 (range, 3–161) months. No systemic chemotherapy was administered before or during SBRT in any patient. Patients and tumor characteristics are summarized in Table 1.

**Table 1.** Patient and tumor characteristics

Patients (*n* = 66)	
Age in years, median (range)	71 (27–87)
Sex (male/female)	44/22
Performance status (0/1/2)	48/17/1
Primary cancer, *n*	
Lungs	31
Colorectal	13
Head and neck	10
Others	12
Primary treatment, *n*	
Surgery	52
Definitive radiotherapy	14
DFI in months	
≤30	31
>30	35
Number of targets (1/2)	56/10
Tumors (*n* = 76)	
Tumor size in cm	
≤3	70
>3	6
Histologic type of oligo-recurrence	
Adenocarcinoma	24
SCC	9
Unknown	43
BED_10_ in Gy	
<100	18
≥100	58

BED = biologically effective dose, SCC = squamous cell carcinoma.

This study was approved by the institutional review board of our institution and written informed consent was obtained from all patients.

### Treatment procedure

The details of the SBRT procedure performed at our institution have been described previously [5]. The SBRT plan was created with a three-dimensional (3D) radiotherapy treatment-planning (RTP) system (XiO, ELEKTA, Stockholm, Sweden). If the respiratory movement of the tumor was <10 mm, as confirmed by fluoroscopy, a treatment-planning CT scan was obtained without breath holding, using a CT-simulator combined with an X-ray simulator system (Aquilion, Toshiba Medical Systems Co. Ltd, Tokyo, Japan) until August 2008. Thereafter, the Discovery ST Elite (GE Healthcare, USA) 4D CT system was used with a real-time position management system (RPM gating system, Varian Medical Systems, USA) and at a 2.0-mm thickness. If the respiratory movement of the tumor was ≥10 mm, a CT scan was performed with breath holding, using a respiratory-monitoring apparatus (Abches, APEX Medical Inc., Tokyo, Japan).

The target margins were defined as follows: the clinical target volume (CTV) was equal to the gross tumor volume (GTV) delineated on CT images displayed with a window level of −300 Hounsfield units (HU) and a window width of 1700 HU; the internal target volume was equal to the CTV plus a 0–10-mm margin based on the tumor respiratory movement, as confirmed with the X-ray simulator or 4D CT; the planning target volume (PTV) was the CTV plus a 5-mm margin in all directions, according to the set-up accuracy. A 5-mm leaf margin was also taken around the PTV.

Irradiation was performed with 10-MV X-ray beams from a linear accelerator (EXL-20TP, Mitsubishi Electric Co. Ltd, Tokyo, Japan) until 2011 and thereafter 6-MV X-ray beams from a linear accelerator (Clinac iX, Varian Medical Systems, USA) in three non-coplanar and three coplanar static ports.

Calculation of the treatment dose was performed using the Clarkson method until October 2009, and thereafter using the Superposition method with 3D-RTP corrected for inhomogeneity. The median isocentric dose was 50 (range, 45–60) Gy administered in a median of 5 (range, 5–9) fractions. The median overall treatment time was 8 (range, 5–22) days, and the median biologically effective dose, assuming an α/β ratio of 10 Gy (BED_10_), was 100 (range, 85.5–120) Gy.

### Follow-up and statistics

The study endpoints were overall survival (OS), disease-free survival (DFS), local control (LC) and toxicity. Follow-up CT scans were obtained at 3–6-month intervals and were used to assess tumor control and toxicity. The patients were also periodically monitored by medical examinations performed during and after treatment. LC was defined as a lack of any significant tumor regrowth on follow-up CT. DFS was defined as a lack of survival and a lack of any recurrence at any sites. The pattern of failure was classified as the second oligo-recurrence or multiple metastases, according to Niibe's criteria for oligo-recurrence [6]. Toxicities were assessed according to the Common Terminology Criteria for Adverse Events, Version 4.0.

Survival estimates were calculated from the first date of SBRT using the Kaplan–Meier method, and statistical differences were determined using the log-rank test. Age (<75 vs ≥75 years), sex (male vs female), performance status (0 vs 1–2), number of targets (1 vs 2), primary lesion (colorectal vs others), histologic type of primary lesion (adenocarcinoma vs squamous cell carcinoma), DFI (≤30 vs >30 months), tumor size (≤3 vs >3 cm), BED_10_ (<100 vs ≥100 Gy) and histologic type of oligo-recurrence (adenocarcinoma vs squamous cell carcinoma vs not available) were all entered into the log-rank test. Statistical significance was defined at *P* < 0.05. All analyses were performed using the IBM SPSS statistics version 22.0 software package (SPSS Inc., Chicago, IL, USA).

## RESULTS

### Survival and LC

**Fig. 1.**
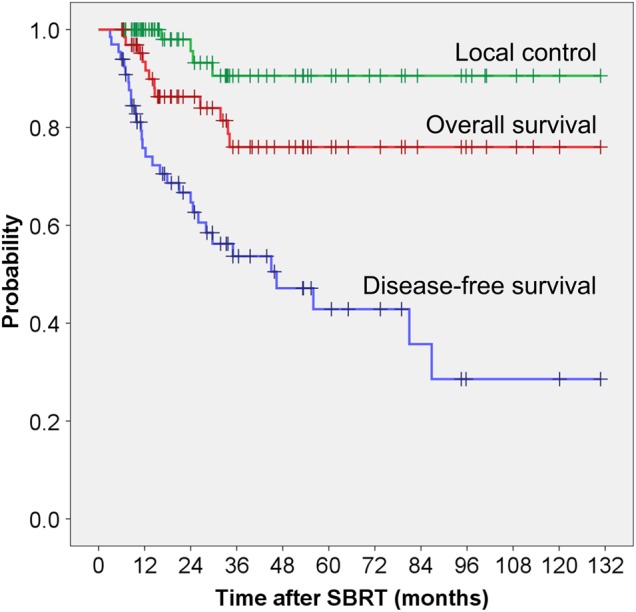
Kaplan–Meier curves for overall survival, disease-free survival, and local control.

The median follow-up time was 31.7 months for all patients, and 36.5 months for the surviving patients. In total, 12 patients (18.2%) died during the follow-up period of 4.3–130 months. The remaining 54 patients were alive, and 28 patients (42.4%) were still alive after >3 years. The causes of death for the 12 patients who died during follow-up were as follows: disease progression, 10; chronic renal failure, 1; and cardiovascular accident (not related to SBRT), 1. The 3-year rates of OS, DFS and LC were 76.0%, 53.7% and 90.6%, respectively (Fig. 1).

### Pattern of failure

Although regrowth of the SBRT target lesion was observed in four patients, no patients experienced local recurrence at the primary lesion site. Distant metastases and/or regional lymph node metastases were observed in 27 patients (Table 2).

**Table 2.** Pattern of failure

	Number of patients	
	Oligo-recurrence	Multiple metastases
Site	(*n* = 20)	(*n* = 11)
Target lesion for SBRT	4	0
Distant only	11	9
Regional only	3	0
Distant and regional	2	2

The sites of and numbers of patients with distant metastases after SBRT were as follows: lungs only, 17; lungs with other sites (bone or brain), 4; and other sites including bone, brain and liver, 3. Accordingly, the most frequent site of distant metastases was the lungs (87.5%). Among the 27 patients with distant metastases and/or lymph node metastases, 16 (59.2%) were judged as having a second oligo-recurrence. Among the 16 patients, 12 who developed new lung metastases were treated with salvage SBRT at 50 Gy in 5 fractions, and all 12 tumors were controlled without disease progression. Salvage surgery was also attempted for two patients (both with colorectal cancer) following regrowth of the SBRT target lesion at 24 and 30 months after SBRT. These patients were still alive without any complications after surgery. Conversely, 11 patients who had multiple metastases were treated with palliative radiotherapy or chemotherapy or best supportive care. Six of these patients had died from disease progression at the last follow-up.

### Toxicity

No patients experienced acute toxicity from treatment. Grade 2 radiation pneumonitis was identified in three patients (4.5%). No other late adverse events of ≥Grade 2 were reported.

### Prognostic factors

We also analyzed differences in patient survival stratified by age, sex, performance status (PS), number of targets, primary cancer, histologic type of primary lesion, DFI, tumor size, and BED for OS, DFS and LC.

**Fig. 2.**
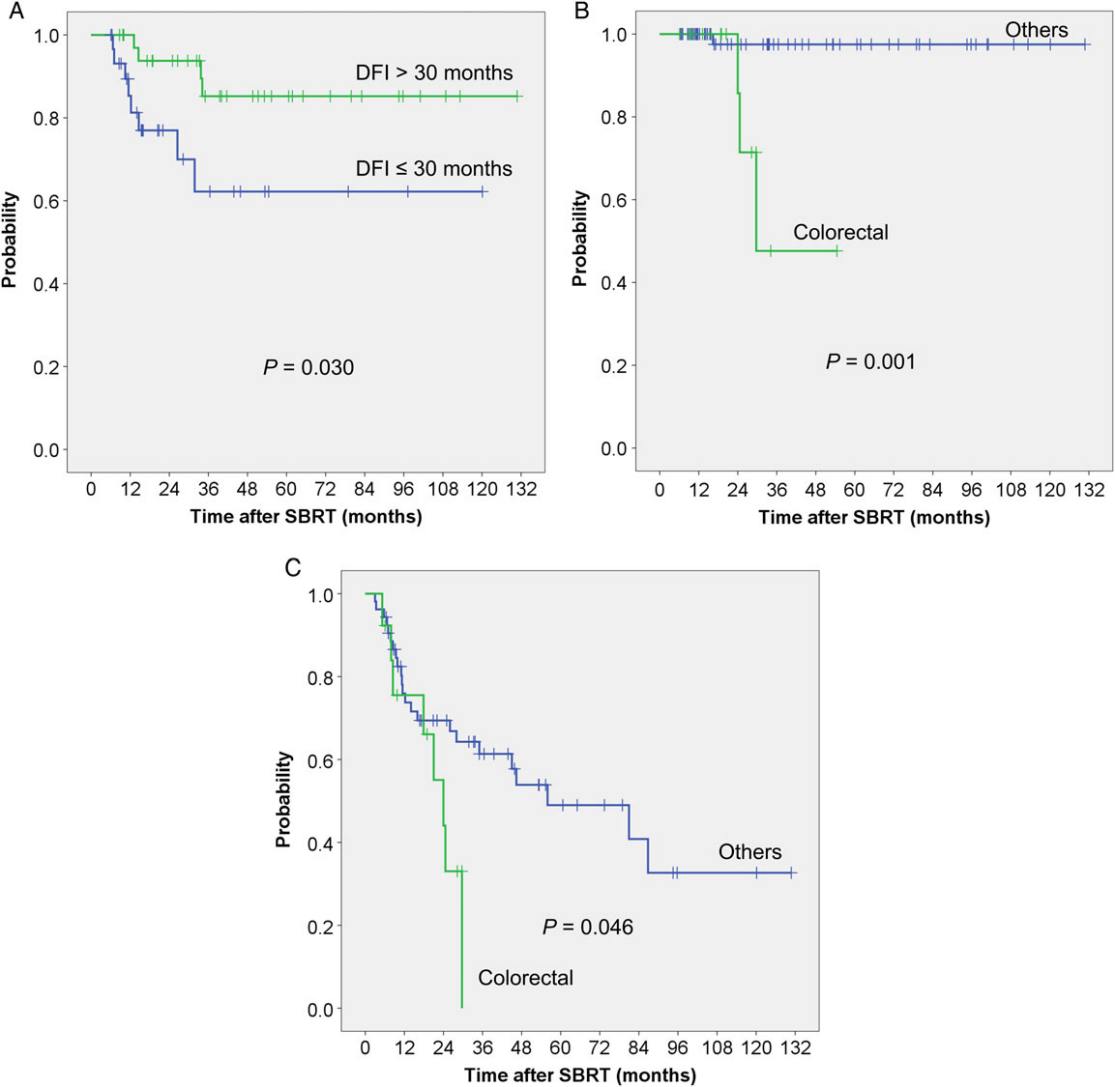
(A) Overall survival (B) Local control (C) Disease-free survival.

DFI was a significant prognostic factor for OS (Table 3). Patients with DFI ≤30 months had a worse prognosis compared with those with DFI >30 months (Fig. 2A). However, DFI did not show significant differences for DFS (Table 3) or LC (Table 4). Oligo-recurrence from colorectal cancer had worse outcomes for LC (Fig. 2B) and DFS (Fig. 2C) compared with those of other primary cancer sites. Other factors such as age, sex, PS, number of targets, histologic type of primary lesion, tumor size, and BED_10_ were not significant factors.

**Table 3.** Three-year overall survival and disease-free survival for all patients

Characteristics	(*n*)	% 3-year OS	*P*-value	% 3-year DFS	*P*-value
Age in years					
<75	46	74.4	0.658	51.5	0.500
≥75	20	80.1		56.6	
Sex					
Male	44	72.8	0.445	55.3	0.903
Female	22	82.0		51.4	
PS					
PS0	48	74.9	0.955	56.2	0.257
PS1-2	18	80.7		46.5	
Number of targets					
1	56	77.8	0.599	54.1	0.748
2	10	57.1		53.3	
Primary lesion					
Colorectal	13	60.6	0.932	0	0.046
Others	53	76.6		61.4	
Histologic type of primary lesion					
Adenocarcinoma	45	79.8	0.315	51.7	0.993
SCC	21	65.1		59.6	
DFI in months					
≤30	31	62.2	0.030	40.4	0.111
>30	35	85.2		64.4	

DFI = disease-free interval, PS = performance status, SCC = squamous cell carcinoma.

**Table 4.** Three-year local control for all tumors

Characteristics	*n*	% 3 years LC	*P*-value
Tumor size in cm			
≤3	70	90.0	0.633
>3	6	100	
BED_10_ in Gy			
<100	18	100	0.154
≥100	58	85.9	
DFI in months			
≤30	35	100	0.191
>30	41	86.8	
Histologic type of oligo-recurrence			
Adenocarcinoma	24	79.6	0.188
SCC	9	100	
Unknown	43	96.0	
Primary cancer			
Colorectal	15	47.6	0.001
Others	61	97.5	

BED = biologically effective dose, DFI = disease-free interval, SCC = squamous cell carcinoma.

## DISCUSSION

In this patient population, the 3-year rates of LC, OS and DFS were 90.6%, 76.0% and 53.7%, respectively. No severe late adverse events of ≥Grade 3 were identified. Patients with a DFI > 30 months had significantly greater OS compared with those with a DFI ≤30 months. Patients with oligo-recurrence from colorectal cancer had significantly worse LC and DFS outcomes compared with those with other primary cancer sites. Although distant metastases and/or regional recurrence after SBRT were observed in 41% of patients, 59% of patients were judged as having a second oligo-recurrence, and salvage SBRT as a local treatment was successful for patients with second oligo-recurrence in the lungs.

### Survival outcome

There are several studies reporting favorable outcomes following SBRT treatment of oligo-recurrence in the lungs. Inoue *et al.* [7] reported the results of SBRT performed on 22 patients with lung oligo-recurrence. With a median follow-up period of 25 months, the 3-year rates of LC and OS were 100% and 72%, respectively. In a study of SBRT treatment of 42 patients with lung oligo-recurrence, Takahashi *et al.* [8] reported that with a median follow-up of 20 months, the 2-year rates of LC and OS were 87% and 65%, respectively. In contrast, the survival of Stage IV non–small-cell lung cancer (NSCLC) is highly limited. In a recent review, the survival outcomes for Stage IV NSCLC treated with thoracic 3D radiotherapy combined with chemotherapy, Su *et al.* [9] reported that the 3-year OS was only 12.9%; however, radiation doses of >63 Gy and metastasis to a single site was associated with better OS. These findings suggest that LC of the primary cancer plays an important role in prolonging the survival of patients with Stage IV lung cancer. In our study, over a median follow-up period of 36 months, all primary cancers were controlled, and the 3-year rates of LC and OS were comparable with the series of oligo-recurrence; however, their OS was better than survival with Stage IV lung cancer.

### Importance of primary cancer status

The status of the primary cancer also plays an important role in prolonging the survival of patients with oligometastases and oligo-recurrence. As was first noted by Niibe *et al.* [6], oligometastases with primary lesions controlled is called ‘oligo-recurrence’, and patients with oligo-recurrence can be treated using local therapy to improve patient survival.

The importance of the primary lesion status has been previously reported for other sites of oligometastases and oligo-recurrence. Jingu *et al.* [10] investigated the long-term benefits of chemoradiotherapy for solitary lymph node metastasis after curative resection of esophageal cancer in 35 patients. Based on the that study, ∼40% of patients with solitary lymph node metastasis after curative resection for esophageal cancer have a chance of long-term survival of >5 years after definitive chemoradiotherapy. In our study, >40% patients with oligo-recurrence in the lungs with a controlled primary cancer had a chance of long-term survival of >3 years. Furthermore, for the patients with a DFI >30 months, the 3-year rate of OS reached 85% in our patient population.

### DFI

Several reports suggest prolonged survival with a longer DFI. However, the value of DFI has not been standardized and varies between 24 and 36 months among studies. Norihisa *et al.* [11] reported clinical results for 34 patients (the majority with lung cancer) with oligometastatic lung tumors after SBRT and suggested that patients with a DFI >36 months had significantly greater OS. Similar findings were reported for patients (the majority with lung cancer) with oligo-recurrence in the lungs after SBRT (a DFI of >36 months [7] and, in another study, a DFI of >31.9 months [8]). On the other hand, Kanzaki *et al.* [12] studied patients with pulmonary metastasectomy from renal cell carcinoma and demonstrated that a DFI ≥24 months was associated with a 5-year survival of 58%, but that the survival of patients with a DFI <24 months decreased to 26%. Because the type of primary tumor generally differs in each study, the predictive value of DFI has not been standardized.

### Oligo-recurrence from colorectal cancer

The type of primary cancer is also an important issue. Oligo-recurrence in the lungs following primary colorectal cancer has generally been considered a worse prognostic factor for LC and DFS than other primary cancers. Hamamoto *et al.* [13] evaluated factors affecting the LC of SBRT with a total dose of 48 Gy in four fractions for lung tumors, including primary lung cancer (*n* = 128 patients) and metastatic lung tumors (colorectum, *n* = 14; other sites, *n* = 17) and observed that the 2-year rates of LC for primary lung cancer and metastatic lung tumors were 87% and 50%, respectively (*P* = 0.0027). Takeda *et al.* [14] reported the outcomes for patients with oligometastatic lung tumors from colorectal cancer (*n* = 21 tumors) and other primary cancers (*n* = 23) treated with SBRT of 50 Gy in five fractions. The 2-year rates of LC for colorectal oligometastases and all other origins were 72% and 94%, respectively (*P* < 0.05). Moreover, a difference in LC between oligometastatic lung tumors from colorectal cancer and other primary cancers after particle beam radiotherapy was reported by Sulaiman *et al.* [15]. The basis for reduced LC rates for oligometastases/oligo-recurrence from colorectal cancer than for other primary cancers is unclear. Therefore, further investigation of the differences in LC between colorectal cancers and other primary cancers is required. Currently, we have initiated a prospective study with respect to LC after SBRT using dual-energy spectral CT imaging [16], and we will report the results in the future.

### Pattern of failure

Although oligo-recurrence in the lungs is controlled after SBRT, distant metastases are likely to occur. In our study, the most frequent sites of recurrence after SBRT were distant metastases, particularly lung metastases, because the lungs are the primary venous drainage organ for the entire body, except for the gastrointestinal tract. Similar findings have been reported in patients with primary lung cancer treated by SBRT. Bradley *et al.* [17] reviewed the records for a total of 91 patients with early-stage NSCLC and observed that the predominant pattern of failure was the development of distant metastasis or second lung cancer. Moreover, Yamamoto *et al.* [18] investigated the clinical results for oligo-recurrence in the lungs by carbon ion radiotherapy and reported that new lesions appeared at other sites, including the lungs, bone and brain, in 60% of patients. Therefore, careful follow-up is necessary for patients with lung oligo-recurrence who are administered local treatment.

On the other hand, Milano *et al.* [19] reported outcomes for patients with oligometastases who underwent two or more curative-intent stereotactic radiotherapy courses and concluded that patients treated with RT for oligometastatic disease can be offered additional courses of radiotherapy to address the interval development of limited metastases and/or locally recurrent disease. Onishi *et al.* [20] also emphasized the importance of local aggressive radiotherapy for patients with repeated oligo-recurrence in multiple organs. In our study, ∼60% of the patients developed recurrence at a site outside the radiation field and were judged as having a second oligo-recurrence. Salvage SBRT was successful in treating patients with a second oligo-recurrence in the lungs.

### Limitations

This study has several limitations. First, it was a retrospective review with a limited number of patients and limited follow-up. Second, it was difficult to distinguish between oligo-recurrence and a second primary lung cancer, because oligo-recurrence in the lungs of a patient with primary NSCLC might represent second primary lung cancers. Third, we treated oligo-recurrence in the lungs from various primary cancers using different therapies. Finally, patients were treated using various fractionation schedules and treatment modalities. However, SBRT can achieve excellent LC with minimal toxicities, regardless of the fractionation schedule for patients with oligo-recurrence in the lungs. Moreover, long-term survival for >3 years can be expected in 42.4% of the patients in our study.

## CONCLUSIONS

SBRT for oligo-recurrence in the lungs achieved acceptable tumor control with minimal toxicities. A longer DFI from initial treatment to SBRT and having a non-colorectal primary cancer were both related to more favorable outcomes. Although second lung metastases occurred after SBRT, additional rounds of SBRT are successful for patients with a second oligo-recurrence in the lungs. Additional follow-up and a larger patient population are required to confirm these favorable outcomes.

## FUNDING

Funding to pay the Open Access publication charges for this article was provided by Hirosaki University.

## References

[1] Hellman S Weichselbaum RR Oligometastases. J Clin Oncol 1995;13:8–10. 7799047 10.1200/JCO.1995.13.1.8

[2] Lewis SL Porceddu S Nakamura N Definitive stereotactic body radiotherapy (SBRT) for extracranial oligometastases: an international survey of >1000 radiation oncologists. *Am J Clin Oncol* 2015. http://www.ncbi.nlm.nih.gov/pubmed/25647831 (2 March 2015, date last accessed). 10.1097/COC.0000000000000169 25647831

[3] Niibe Y Kazumoto T Toita T Frequency and characteristics of isolated para-aortic lymph node recurrence in patients with uterine cervical carcinoma in Japan: a multi-institutional study. Gynecol Oncol 2006;103:435–8. 16677691 10.1016/j.ygyno.2006.03.034

[4] Niibe Y Kenjo M Kazumoto T Multi-institutional study of radiation therapy for isolated para-aortic lymph node recurrence in uterine cervical carcinoma: 84 subjects of a population of more than 5,000. Int J Radiat Oncol Biol Phys 2006;66:1366–9. 17126206 10.1016/j.ijrobp.2006.07.1384

[5] Aoki M Abe Y Kondo H Clinical outcome of stereotactic body radiotherapy of 54 Gy in nine fractions for patients with localized lung tumor using a custom-made immobilization system. Radiat Med 2007;25:289–94. 17634882 10.1007/s11604-007-0141-7

[6] Niibe Y Hayakawa K Oligometastases and oligo-recurrence: the new era of cancer therapy. Jpn J Clin Oncol 2010;40:107–11. 20047860 10.1093/jjco/hyp167 PMC2813545

[7] Inoue T Katoh N Onimaru R Clinical outcomes of stereotactic body radiotherapy for patients with lung tumors in the state of oligo-recurrence. Pulm Med 2012;2012:369820. 22848816 10.1155/2012/369820 PMC3399335

[8] Takahashi W Yamashita H Niibe Y Stereotactic body radiotherapy for metastatic lung cancer as oligo-recurrence: an analysis of 42 cases. Pulm Med 2012;2012:454107. 23094150 10.1155/2012/454107 PMC3472526

[9] Su SF Hu YX Ouyang WW The survival outcomes and prognosis of stage IV non-small-cell lung cancer treated with thoracic three-dimensional radiotherapy combined with chemotherapy. Radiat Oncol 2014;9:290. 25518882 10.1186/s13014-014-0290-7 PMC4278263

[10] Jingu K Ariga H Nemoto K Long-term results of radiochemotherapy for solitary lymph node metastasis after curative resection of esophageal cancer. Int J Radiat Oncol Biol Phys 2012;83:172–7. 22079727 10.1016/j.ijrobp.2011.06.1978

[11] Norihisa Y Nagata Y Takayama K Stereotactic body radiotherapy for oligometastatic lung tumors. Int J Radiat Oncol Biol Phys 2008;72:398–403. 18374506 10.1016/j.ijrobp.2008.01.002

[12] Kanzaki R Higashiyama M Fujiwara A Long-term results of surgical resection for pulmonary metastasis from renal cell carcinoma: a 25-year single-institution experience. Eur J Cardiothorac Surg 2011;39:167–72. 20591686 10.1016/j.ejcts.2010.05.021

[13] Hamamoto Y Kataoka M Yamashita M Local control of metastatic lung tumors treated with SBRT of 48 Gy in four fractions: in comparison with primary lung cancer. Jpn J Clin Oncol 2010;40:125–9. 19825814 10.1093/jjco/hyp129

[14] Takeda A Kunieda E Ohashi T Stereotactic body radiotherapy (SBRT) for oligometastatic lung tumors from colorectal cancer and other primary cancers in comparison with primary lung cancer. Radiother Oncol 2011;101:255–9. 21641064 10.1016/j.radonc.2011.05.033

[15] Takeda A Sanuki N Kunieda E Role of stereotactic body radiotherapy for oligometastasis from colorectal cancer. World J Gastroenterol 2014;20:4220–9. 24764660 10.3748/wjg.v20.i15.4220 PMC3989958

[16] Aoki M Takai Y Narita Y Correlation between tumor size and blood volume in lung tumors: a prospective study on dual-energy gemstone spectral CT imaging. J Radiat Res 2014;55:917–23. 24829253 10.1093/jrr/rru026 PMC4202284

[17] Bradley JD Naqa IE Drzymala RE Stereotactic body radiation therapy for early-stage non-small-cell lung cancer: the pattern of failure is distant. Int J Radiat Oncol Biol Phys 2010;77:1146–50. 19800181 10.1016/j.ijrobp.2009.06.017

[18] Yamamoto N Nakajima M Tsujii H Carbon ion radiotherapy for oligo-recurrence in the lung. Pulm Med 2013;2013:219746. 23431437 10.1155/2013/219746 PMC3568897

[19] Milano MT Philip A Okunieff P Analysis of patients with oligometastases undergoing two or more curative-intent stereotactic radiotherapy courses. Int J Radiat Oncol Biol Phys 2009;73:832–7. 18760543 10.1016/j.ijrobp.2008.04.073

[20] Onishi H Ozaki M Kuriyama K Stereotactic body radiotherapy for metachronous multisite oligo-recurrence: a long-surviving case with sequential oligo-recurrence in four different organs treated using locally radical radiotherapy and a review of the literature. Pulm Med 2012;2012:713073. 23150822 10.1155/2012/713073 PMC3486341

